# A case report of Castleman disease variant of POEMS syndrome presenting with prominent polyserositis and renal impairment

**DOI:** 10.3389/fmed.2025.1537944

**Published:** 2025-06-16

**Authors:** Congcong Min, Ailing Liu, Yushuang Xu, Yanan Yu, Yonghong Xu, Tao Mao, Xueli Ding

**Affiliations:** Department of Gastroenterology, the Affiliated Hospital of Qingdao University, Qingdao, China

**Keywords:** POEMS syndrome, Castleman disease, M protein, renal impairment, case report

## Abstract

**Background:**

POEMS syndrome is a rare hematologic disorder related to plasma cell dyscrasia. The Castleman disease variant of POEMS syndrome is extremely rare and often misdiagnosed. In this study, we aim to present a noteworthy case of POEMS syndrome mainly manifested as multiple pleural effusion and renal impairment without M protein.

**Case presentation:**

A 47-year-old woman was admitted to the hospital with a 7-month history of lower extremity edema and 3 months of abdominal distension. Computed tomography revealed poly-serosal effusion and hepatosplenomegaly, while ultrasound showed multiple superficial lymphadenopathies. Serum protein electrophoresis and bone biopsy indicated no evidence of monoclonal plasma cell proliferation. Pathological results obtained from lymph node biopsy revealed Castleman disease (CD). The patient was ultimately diagnosed with the Castleman disease variant of POEMS syndrome without M protein. Renal function gradually declined in the later stages of the disease. After transferring to another hospital, the patient received a VPD chemotherapy regimen (Pomalidomide, Bortezomib, and Dexamethasone) and hemodialysis. Effusions in multiple serosal cavities were reduced, and renal function improved significantly following active treatment.

**Conclusion:**

POEMS syndrome without M protein is often misdiagnosed as other conditions. In patients presenting with multiple systemic manifestations, the possibility of POEMS syndrome or CD should be considered.

## 1 Introduction

POEMS syndrome is an uncommon paraneoplastic disorder arising from plasma cell neoplasms, marked by polyneuropathy, organomegaly, endocrinopathy, M protein, and skin changes. A 2003 survey conducted in Japan estimated the prevalence of POEMS syndrome at approximately 0.3 per 100,000 (1). The pathogenesis mechanism of POEMS syndrome remains unclarified, but it may be linked to cytokine imbalances, including transforming growth factor β1 (TGFβ1), interleukin-6 (IL-6), and serum vascular endothelial growth factor (VEGF) (2). Major diagnostic criteria for POEMS syndrome include multiple peripheral neuropathy and monoclonal plasma cell proliferation. Other major criteria are sclerotic bone lesions, increased VEGF, and Castleman disease (CD). Minor criteria encompass organomegaly, endocrine abnormalities, specific skin changes, papilledema, extravascular volume overload, and thrombocytosis (2). Due to its rarity and diverse clinical manifestations, POEMS syndrome is often misdiagnosed.

CD is a rare lymphoproliferative disorder, also known as angiofollicular hyperplasia. Based on clinical presentation and disease course, CD is divided into two subtypes: unicentric CD (UCD) and multicentric CD (MCD). MCD is further classified into HHV-8 positive and idiopathic forms, depending on the presence of HHV-8 infection. Pathologically, CD can be categorized into hyaline vascular, plasma cell, and mixed subtypes (3, 4). The etiology and pathogenesis of CD remain unclear. CD has been reported in 11–30% of patients diagnosed with POEMS syndrome who underwent lymph node biopsy (5). In addition to the classical POEMS syndrome, a variant of Castleman disease may present without multiple neuropathies or M protein. This report describes a case of the Castleman disease variant of POEMS syndrome, initially presenting with multi-serosal effusion and edema.

## 2 Case presentation

A 47-year-old woman was admitted to the hospital with a 7-month history of lower extremity edema and 3 months of abdominal distension on October 26, 2022. She also had a 1-year history of resistant hypertension and blurred vision. Seven months prior, she developed bilateral ankle edema without an obvious cause, followed by abdominal distension 3 months ago. However, she did not seek medical attention at that time. Over time, the edema in both lower limbs and abdominal distension worsened, accompanied by nausea, poor appetite, and fatigue. One month ago, she was firstly admitted to a local hospital, where imaging examinations revealed splenomegaly, hepatomegaly, and multiple serous cavity effusions (pleural, abdominal, and pericardial). Cardiac ultrasound indicated moderate pulmonary hypertension (64 mmHg) and mild pericardial effusion. Breast ultrasound showed enlargement of bilateral axillary lymph nodes. Urinalysis indicated urinary protein 2+, but renal function remained normal. Ascitic fluid analysis revealed an exudate. Despite symptomatic treatment with antihypertensive and diuretic therapy, her symptoms persisted without a clear diagnosis. During diuresis or ascitic fluid discharge, she also experienced numbness in the distal limbs. As a result, she was referred to our hospital for further evaluation and treatment.

**FIGURE 1:**
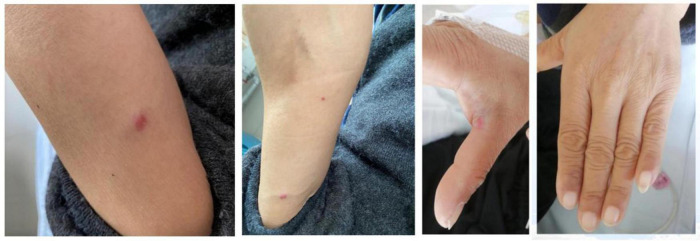
Skin changes-glomeruloid hemangioma and white nails.

The patient had stable vital signs, with a blood pressure of 146/77 mmHg. There was no evident pallor of the palpebral conjunctiva or yellowing of the sclera. Multiple enlarged lymph nodes were palpated bilaterally in the axillary and groin regions, with the largest measuring approximately 4 cm × 0.5 cm. Breath sounds in both lungs were clear, and the heart rate was regular at 77 beats per minute. The abdomen was slightly distended with positive mobile dullness, but no tenderness or rebound pain was noted. The liver and spleen were not palpable below the costal margin. Mild pitting edema was present in both lower limbs. Multiple glomeruloid hemangiomas were visible on the skin of the limbs, and leukonychia was observed on the fingers (Figure 1). The patient also reported bilateral toe numbness. Muscle strength and tension in the extremities were normal, but tendon reflexes in the lower extremities were diminished. Bilateral Babbitt signs were negative.

**FIGURE 2:**
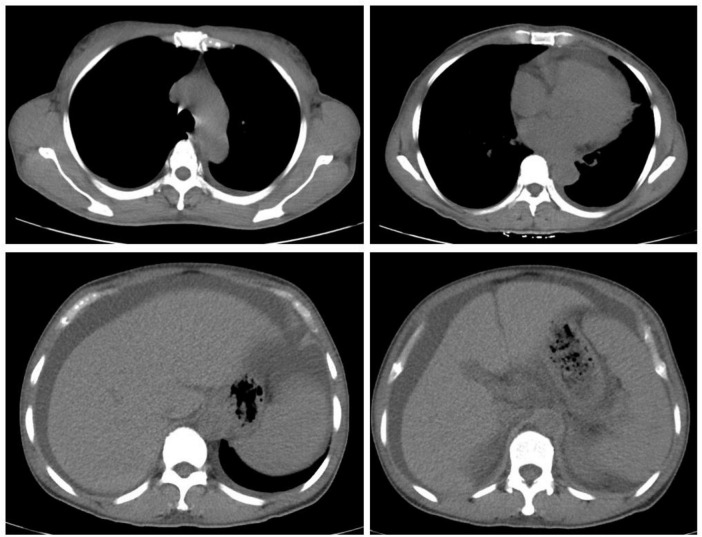
CT revealed hepatosplenomegaly and multiple serous effusion. CT, computed tomography.

Upon admission, the patient’s blood routine showed a hemoglobin concentration of 114 g/L and a platelet count of 361 × 10^9^/L. Blood biochemistry results indicated serum albumin at 32.7 g/L, urea at 25.73 mmol/L, creatinine at 164 μmol/L, fasting blood glucose at 4.49 mmol/L, calcium at 2.00 mmol/L, phosphorus at 1.73 mmol/L and uric acid at 636 umol/L. The coagulation test revealed a D-dimer level of 1320 ng/mL. The erythrocyte sedimentation rate was 29 mm/h. Tumor markers showed CA125 at 91.69 U/mL, while other markers were normal. ANA antibodies, ANCA, ENA spectrum, anti-dsDNA antibody, and anticardiolipin antibodies were all negative. Parathyroid hormone levels were normal. Thyroid function tests showed TSH at 9.220 uIU/mL, FT3 at 2.25 pmol/L, and FT4 at 10.60 pmol/L, indicating clinical hypothyroidism. Cortisol, ACTH, and sex hormones were unremarkable. β2-microglobulin was significantly elevated at 8303 μg/L. The blood immunoglobulin κ/λ light chain ratio was decreased, with urine immunoglobulin κ and λ light chains elevated (130 mg/L and 92.2 mg/L, respectively). However, serum protein electrophoresis and immunofixation did not detect M protein. Urinalysis revealed occult blood 1 + , urine protein 2 + , and urinary microalbumin at 1067.6 mg/L. The 24-h urine protein level was 1.33 g. Chest and abdominal computed tomography revealed (1) Small amounts of pleural effusion bilaterally, increased cardiac shadow, and pericardial effusion; (2) Splenomegaly, hepatomegaly, and moderate fluid accumulation in the abdominal cavity and pelvis (Figure 2). The ascitic fluid was found to be predominantly leaking fluid. A thyroid ultrasound revealed uneven thyroid enlargement and thyroid nodules. Superficial lymph node ultrasound showed multiple enlarged lymph nodes in the bilateral cervical, axillary, and groin regions, with the largest node in the left groin measuring 4.7 × 0.6 cm. Cardiac ultrasound indicated: (1) Left ventricular myocardial hypertrophy; (2) Mild regurgitation of the aortic, mitral, and tricuspid valves; (3) Mild pulmonary hypertension (44 mmHg); (4) Reduced left ventricular diastolic function; (5) Minor pericardial effusion. VEGF was elevated at 266.04 pg/mL (normal range: 0–160 pg/mL), and interleukin-6 was 19.01 pg/mL (normal range: 0–7 pg/mL). Electromyography indicated multiple peripheral nerve lesions with both motor and sensory involvement, more severe in the lower extremities. Bone marrow aspiration revealed 0.02% plasma cells, with a 3 ratio of cLambda to cKappa. Flow cytometric data analysis of bone marrow blood showed positivity of CD38, CD138, CD19, CD81, CD56, CD27 and negativity of CD117. Bone marrow biopsy showed scattered or clustered distribution of plasma cells (approximately 5%) with slightly increased plasma cells expressing Lambda. But there was no evidence of monoclonal plasma cell proliferation. PET-CT showed: (1) Multiple serous cavity effusions (pericardium, bilateral pleural cavity, and abdominal pelvis); (2) Multiple mildly enlarged lymph nodes in the bilateral neck, axillae, retroperitoneum, and groin, with SUVmax of 1.8; (3) Enlarged spleen, considered secondary changes. But there were no any osteosclerotic lesions on PET-CT.

The patient primarily presented with edema, multiple serous effusions (pleural, abdominal, and pericardial), hepatomegaly, splenomegaly, lymphadenopathy, a mild increase in serum creatinine, and skin changes. A departmental discussion and multidisciplinary consultation were conducted on the fifth day after admission. The differential diagnosis included POEMS syndrome, connective tissue disease, malignancy, lymphoma, and multiple myeloma. Connective tissue disease was excluded based on the negative autoantibody results. PET-CT also showed no evidence of solid tumors or lymphoma. Bone marrow aspiration revealed no monoclonal plasma cell proliferation or plasma cell tumors, ruling out multiple myeloma. The patient complained of numbness at the extremities, especially during ascites extraction, prompting electromyography, which revealed multiple peripheral nerve lesions affecting both motor and sensory functions. The motor nerve conduction velocity (MCV) and sensory nerve conduction velocity (SCV) of the median and ulnar nerves are both slowed down. The MCV of the tibial nerve and common peroneal nerve were significantly slowed down and the amplitude of the CAMP wave was remarkably reduced, but F wave was not be measured. Moderate to large spontaneous potentials can be observed in the tibialis anterior muscle, gastrocnemius muscle, and peroneal longus muscle in a resting state, with slightly poor recruitment during vigorous contractions. The patient exhibited polyneuropathy (P), organomegaly (O), endocrinopathy (E), and skin changes (S) but lacked M protein (M). This raised the question of whether the patient could be diagnosed with POEMS syndrome. POEMS syndrome is a rare paraneoplastic syndrome related to plasma cell hyperplasia. It typically presents with these five clinical features, although they may not occur simultaneously.

**FIGURE 3:**
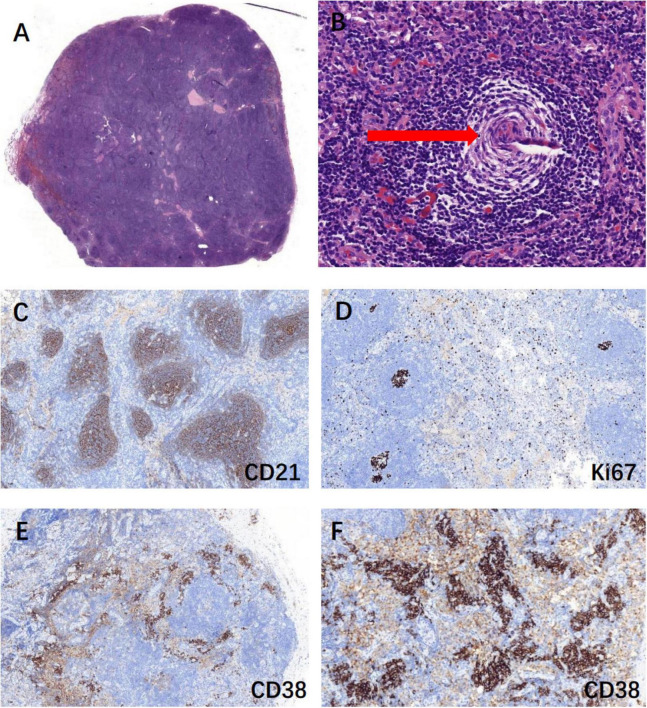
Biopsy of the right inguinal lymph node suggested Castleman disease. **(A)** Lymphoid follicular hyperplasia, H&E stain, 10×; **(B)** onion skin-like lymphoid follicles (red arrow): thickened follicular sheath areas and germinal center atrophy, H&E stain, 100×; **(C)** CD21 positive marked follicular dendritic network, IHC, 100×; **(D)** Ki67-positive rate of 20% in non-germinal centers, IHC, 100×; **(E,F)** CD38 positive showed massive plasma cell infiltration, IHC, 100× & IHC, 200×.

Update on the latest diagnostic criteria for POEMS syndrome in 2023 (6) include: (1) Mandatory major criteria: polyneuropathy and monoclonal plasma cell-proliferative disorder; (2) Other major criteria (at least one required): Castleman disease, sclerosing bone lesions, and VEGF elevation; (3) Minor criteria: organomegaly (such as splenomegaly, hepatomegaly, or lymphadenopathy), extravascular volume overload (e.g., edema, pleural effusion, ascites), endocrinopathy, specific skin changes (e.g., hyperpigmentation, hypertrichosis, glomeruloid hemangiomas, white nails), papilledema, and thrombocytosis/polycythemia. A POEMS syndrome diagnosis requires both mandatory major criteria, at least one primary criterion, and at least one secondary criterion. The Castleman disease variant of POEMS syndrome is diagnosed when there is no evidence of monoclonal plasma cell proliferation. To confirm the diagnosis, we performed a right inguinal lymph node excision biopsy. The pathological examination revealed proliferating lymphoid follicular structures, thickened follicular sheath areas and germinal center atrophy (onion-skin-like lymphoid follicles). Small vessel hyperplasia was observed in some germinal centers, and massive plasma cell infiltration in the interfollicular areas, consistent with Castleman disease (Figure 3). VEGF levels were elevated to more than 200 pg/mL. Ocular B ultrasonography showed vitreous opacity and papilledema. This patient met 1 mandatory major criterion (polyneuropathy), 2 primary criteria (Castleman disease and VEGF elevation), and 6 secondary criteria (organomegaly, extravascular volume overload, endocrinopathy, skin changes, papilledema, and pulmonary hypertension). Therefore, the patient was ultimately diagnosed with the Castleman disease variant of POEMS syndrome, as there was no evidence of monoclonal plasma cell proliferation.

**FIGURE 4:**
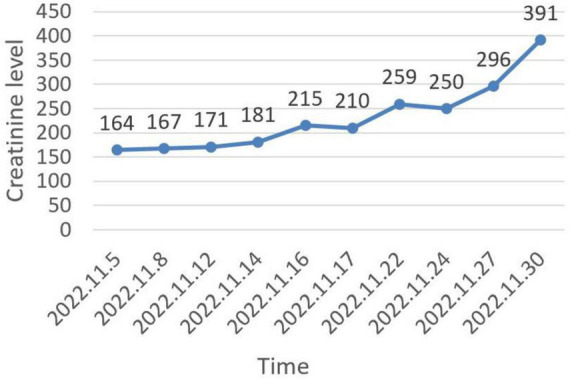
Trend of creatinine level changes during hospitalization.

The patient was initially treated with diuretics, albumin infusion, and intermittent drainage of ascites. Antihypertensive treatment included nifedipine controlled-release tablets and irbesartan hydrochlorothiazide. However, the ascites did not significantly reduce. Over the course of 26 days, creatinine progressively increased to 391 μmol/L (Figure 4), and urine output decreased to 600–1000 mL/day. Due to the presence of ascites, renal biopsy was not recommended. The patient was subsequently referred to a superior hospital for further strategies. VEGF was reviewed at 188.42 pg/mL, IL-6 at 24.36 pg/mL, and HHV-8 DNA was normal. The diagnosis of Castleman disease variant of POEMS syndrome was still maintained. As the patient’s urine volume decreased further to 50–300 mL/day, hemodialysis was initiated. The patient also started chemotherapy with the VPD regimen (pomalidomide 4 mg daily for 3 weeks, Dexamethasone 20 mg once a week, and Bortezomib 1.5 mg once a week). Over the past year, she continued regular chemotherapy and weekly hemodialysis. Ascites significantly reduced, and serum creatinine decreased to 126 μmol/L by the follow-up on September 30, 2024. However, the muscle strength in both lower extremities was notably affected, and she was unable to walk independently.

## 3 Discussion

POEMS syndrome is a rare paraneoplastic syndrome resulting from an abnormal proliferation of plasma cells, first described by Crow in 1956 (7). The incidence is approximately 3 per 100,000, with a male predominance (8). The pathogenesis of POEMS syndrome remains largely unknown, though the overproduction of pro-inflammatory cytokines such as TGF-β1, IL-6, and VEGF play a key role in its development (9, 10). VEGF is the cytokine most closely correlated with disease activity, making it valuable for both diagnosis and treatment response evaluation (6). POEMS syndrome predominantly presents with demyelinating peripheral neuropathy and multiple systems involvement. A thorough history, physical examination, and appropriate testing are crucial for diagnosing POEMS syndrome, which is often misdiagnosed due to its overlap with other disorders.

CD is a heterogeneous group of lymphoproliferative disorders, also known as giant lymph node disease or vascular follicular lymph node hyperplasia. While it shares common lymphoid tissue pathological features, its etiology and clinical manifestations are diverse (4). CD can manifest as asymptomatic lymph node enlargement or multiple system damage. It is divided into UCD and MCD based on the clinical presentation and lymph node distribution. UCD typically involves a single enlarged lymph node with mild symptoms, and surgical excision is often curative (4). MCD is further subdivided into HHV-8 positive and HHV-8 negative forms (11). HHV-8 negative MCD can be further categorized into asymptomatic MCD (aMCD) and idiopathic MCD (iMCD), with the latter often associated with systemic symptoms and/or organ damage, such as in POEMS syndrome. Lymph node biopsy remains the gold standard for diagnosing CD. Pathologically, CD is classified into three subtypes: hyaline vascular subtype (HV-CD), plasma cell subtype (PC-CD), and mixed type CD. It is estimated that approximately 11%–30% of patients diagnosed with POEMS syndrome who undergo lymph node biopsy show evidence of concurrent CD (5).

This case presented with prominent features of multiple pleural effusions and peripheral edema at the early onset of the disease. The differential diagnosis should first focus on the etiology of the multiple pleural effusions. The nature of the ascites was between transudate and exudate. In the absence of sufficient evidence of infection, autoimmune diseases, or solid tumors, lymphoproliferative disorders should be considered, especially due to the presence of multiple lymphadenopathies. Additionally, the patient exhibited organ enlargement, extravascular volume overload (peripheral edema, pleural effusion, ascites, and pericardial effusion), endocrine abnormalities, and skin changes, which met multiple secondary criteria for POEMS syndrome. Electromyography further confirmed multiple peripheral nerve lesions. VEGF was markedly elevated to more than 200 pg/mL, while HHV-8 DNA was normal. Due to retroperitoneal and multiple superficial lymph node enlargements (in the bilateral neck, axilla, and groin) on PET-CT and ultrasound, a right inguinal lymph node resection biopsy was performed. Pathological examination confirmed CD. Based on the clinical presentations and pathological features, the patient was classified as HHV-8 negative iMCD. The patient met 1 mandatory major criterion, 2 primary criteria, and multiple secondary criteria but showed no evidence of M protein or monoclonal plasma cell proliferation. As a result, she was not diagnosed with classic POEMS syndrome. According to the updated 2021 POEMS syndrome diagnostic criteria, risk stratification, and management guidelines (2), the patient was classified as CD variant of POEMS syndrome due to the absence of monoclonal plasma cell proliferation. Multiple pleural effusions are not uncommon in patients with MCD. Zhang et al. (12) and Liu et al. (13) reported that 18.8% and 23% of patients with MCD developed ascites and/or pleural effusions, respectively. When encountering patients with unexplained multiple pleural effusions, it is recommended to differentiate it from CD by conducting a thorough medical history inquiry and superficial lymph node examination, which can provide diagnostic clues and aid in clinical diagnosis.

During hospitalization, the patient received antibiotics, diuretics, and ascites drainage to alleviate symptoms. However, over the course of more than 20 days, the serum creatinine level gradually increased to approximately 400 μmol/L, accompanied by a slight decrease in urine output. The renal dysfunction was believed to be associated with the progression of the primary disease, the Castleman disease variant of POEMS syndrome. However, a renal biopsy could not be performed due to the presence of ascites. The patient was transferred to another hospital for further therapy.

Renal impairment is a common complication of POEMS syndrome. Edema is the most frequent clinical manifestation of renal injury, often accompanied by mild to moderate proteinuria, while serum creatinine levels typically remain normal (14). Approximately 4% of patients develop renal failure as a preterminal event (8). In a Chinese study, 22.4% of patients had a baseline glomerular filtration rate (eGFR) below 60 mL/min/1.73 m^2^ (15). Renal involvement occurs more frequently in patients with co-existing CD (6). Renal dysfunction can be reversed in 66% of patients who receive effective therapy, with a shorter interval between symptom onset and treatment (OR 0.059, *P* = 0.043) and VEGF remission (OR 15.958, *P* = 0.050) showing significant associations in multivariate analysis (15). Patients with severe renal impairment (eGFR < 30 mL/min/1.73 m^2^) had poorer survival compared to those without renal impairment (15). A good renal response may indicate an improved prognosis. In POEMS syndrome, renal histopathological findings are varied, with renal damage incidence reported between 8.5% and 54% in recent studies (16, 17). Renal biopsy is the most reliable method for diagnosing renal damage caused by POEMS syndrome or CD, typically revealing endothelial cell proliferation, subendothelial loosening, mesangial dissolution, and related changes on light and electron microscopy (6). Common pathological findings include crescentic nephritis, thrombotic microangiopathy (TMA), and light chain amyloidosis (18). In our case, severe renal impairment (eGFR = 16 mL/min/1.73 m^2^) developed as the disease progressed, ultimately requiring hemodialysis. Due to the presence of significant ascites, a renal biopsy could not be performed, limiting the ability to assess the degree and specific pathology of the renal impairment.

Currently, there is no standardized chemotherapy regimen for POEMS syndrome. Over recent decades, conventional agents such as melphalan combined with corticosteroids have proven effective and safe. Autologous hematopoietic stem cell transplantation (ASCT) remains an option for high-risk patients who are eligible for transplantation. Although anti-myeloma agents, including Thalidomide, Lenalidomide, and Bortezomib, have shown promising results in treating POEMS syndrome (19, 20). Large-scale and long-term follow-up studies are needed to develop optimal regimens. For patients not undergoing ASCT, the LDex regimen (Lenalidomide with Dexamethasone) is the most widely used regimen (19). Zhao et al. (21) conducted a retrospective study of nearly 350 POEMS syndrome patients, finding the highest response rates with ASCT, followed by Lenalidomide with Dexamethasone, and then melphalan with Dexamethasone. For UCD patients, complete surgical resection is preferred. In MCD, Cetuximab (an IL-6 monoclonal antibody) serves as the first-line treatment for non-severe iMCD patients (22). Other first-line options include the TCP regimen (Thalidomide + Cyclophosphamide + Prednisone) and rituximab-based therapies (22–24). The second-line options consist of the BCD regimen (Bortezomib + Cyclophosphamide + Dexamethasone), VDT regimen (Bortezomib + Dexamethasone + Thalidomide), or combinations with rituximab or cetuximab. In our patient’s case, more than 6 months elapsed from symptom onset to diagnosis, during which the disease progressed rapidly, causing severe renal injury and a poor response to conventional treatments. After referral to another hospital, she received regular hemodialysis (three times a week) and VPD regimen chemotherapy (Pomalidomide + Dexamethasone + Bortezomib). Her symptoms and renal function improved significantly within a short time. As of the follow-up in October 2024, the patient continued with weekly hemodialysis and VPD regimen chemotherapy. Her serum creatinine level had decreased to 126 μmol/L. Despite improvements, she experienced reduced muscle strength in both lower limbs, with relatively slow neurological recovery. The patient remains under regular follow-up.

The prognosis of POEMS syndrome is generally better than that of multiple myeloma. A recent Chinese study involving 621 POEMS syndrome cases reported that the 6-year progression-free survival (PFS) rate for these patients exceeds 50%, with a median survival of 184 months (19). With advancements in treatment strategies, the overall survival rate for POEMS syndrome has significantly improved. Currently, no molecular or genetic risk factors are known to predict overall survival in POEMS syndrome. A previous study revealed that key risk factors include older age, pleural effusion, pulmonary hypertension, low serum albumin and decreased eGFR (6). Chinese researchers have developed a risk nomogram incorporating factors such as age over 50, pleural effusion, an eGFR below 30 mL/min/1.73 m^2^, and pulmonary hypertension (25). In one study, lower VEGF levels were associated with better treatment response (26). POEMS patients with concurrent CD may have a poorer overall survival compared to those without CD (27).

## 4 Conclusion

In summary, POEMS syndrome can affect multiple organs and systems, with diverse clinical manifestations that are often susceptible to misdiagnosis or delayed diagnosis. This study reports a rare case of Castleman disease variant of POEMS syndrome. In patients with multisystem involvement, such as unexplained poly-serosal effusions, renal impairment, and lymph node enlargement, the possibility of POEMS syndrome or CD should be considered. Diagnosis of CD relies on pathological examination, and a complete or partial biopsy of affected lymph nodes is recommended. Early recognition and prompt treatment can improve the outcomes of patients with POEMS syndrome.

## Data Availability

The original contributions presented in this study are included in this article/supplementary material, further inquiries can be directed to the corresponding author.
